# Effects of Fructose or Glucose on Circulating ApoCIII and Triglyceride and Cholesterol Content of Lipoprotein Subfractions in Humans

**DOI:** 10.3390/jcm8070913

**Published:** 2019-06-26

**Authors:** Bettina Hieronimus, Steven C. Griffen, Nancy L. Keim, Andrew A. Bremer, Lars Berglund, Katsuyuki Nakajima, Peter J. Havel, Kimber L. Stanhope

**Affiliations:** 1 Department of Molecular Biosciences, School of Veterinary Medicine, University of California, Davis, CA 95616, USA; 2 Department of Internal Medicine, School of Medicine, University of California, Davis, Sacramento, CA 95817, USA; 3 United States Department of Agriculture, Western Human Nutrition Research Center, Davis, CA 95616, USA; 4 Department of Nutrition, University of California, Davis, CA 95616, USA; 5 Department of Pediatrics, School of Medicine, University of California, Davis, Sacramento, CA 95817, USA; 6 Department of Clinical Laboratory Medicine, Gunma University Graduate School of Medicine, Maebashi, Gunma 371-8510, Japan; 7 Hidaka Hospital, Takasaki, Gunma 370-0001, Japan; 8 General Internal Medicine, Kanazawa Medical University, Kanazawa 920-0265, Japan; 9 Laboratory of Clinical Nutrition and Medicine, Kagawa Nutrition University, Tokyo 350-0288, Japan

**Keywords:** sugar, atherosclerosis risk factors, lipoprotein fractions, TG-rich lipoproteins, clinical studies, LDL, lipid and lipoprotein metabolism, nutrition/carbohydrates

## Abstract

ApoCIII and triglyceride (TG)-rich lipoproteins (TRL), particularly, large TG-rich lipoproteins particles, have been described as important mediators of cardiovascular disease (CVD) risk. The effects of sustained consumption of dietary fructose compared with those of sustained glucose consumption on circulating apoCIII and large TRL particles have not been reported. We measured apoCIII concentrations and the TG and cholesterol content of lipoprotein subfractions separated by size in fasting and postprandial plasma collected from men and women (age: 54 ± 8 years) before and after they consumed glucose- or fructose-sweetened beverages for 10 weeks. The subjects consuming fructose exhibited higher fasting and postprandial plasma apoCIII concentrations than the subjects consuming glucose (*p* < 0.05 for both). They also had higher concentrations of postprandial TG in all TRL subfractions (*p* < 0.05, effect of sugar), with the highest increases occurring in the largest TRL particles (*p* < 0.0001 for fructose linear trend). Compared to glucose consumption, fructose consumption increased postprandial TG in low-density lipoprotein (LDL) particles (*p* < 0.05, effect of sugar), especially in the smaller particles (*p* < 0.0001 for fructose linear trend). The increases of both postprandial apoCIII and TG in large TRL subfractions were associated with fructose-induced increases of fasting cholesterol in the smaller LDL particles. In conclusion, 10 weeks of fructose consumption increased the circulating apoCIII and postprandial concentrations of large TRL particles compared with glucose consumption.

## 1. Introduction

The incidence and prevalence of undesirable health outcomes including obesity, type-2 diabetes, cardiovascular disease (CVD), and metabolic syndrome are increasing in developing and developed countries alike, with CVD being the number one cause of death globally [1]. Dietary habits affect cardiometabolic risk [2], but we lack a full understanding of how dietary patterns influence the development of undesirable lipid profiles that lead to metabolic diseases. Understanding the mechanisms that link specific dietary components and patterns to atherogenic dyslipidemia will promote the implementation of dietary policies to reduce CVD risk.

We earlier reported the results from a 10-week intervention trial with women and men (age: 54 ± 8 years; body mass index (BMI): 29.1 ± 2.9 kg/m^2^ (mean ± SD)) who consumed 25% of their energy requirement from fructose- or glucose-sweetened beverages [3]. Despite comparable weight gain in both groups, fructose consumption promoted lipid dysregulation, while glucose consumption did not [3]. Compared with glucose, the consumption of fructose increased the circulating concentrations of postprandial triglycerides (TG), remnant-like particle lipoprotein (RLP)-TG, and RLP-cholesterol (chol), as well as those of fasting total chol, low-density lipoprotein (LDL)-chol, apolipoprotein B (apoB), small dense LDL-chol (sdLDL-chol), and oxidized LDL [3]. Subjects consuming fructose also exhibited increased postprandial hepatic de novo lipogenesis (DNL) and decreased insulin sensitivity compared with subjects consuming glucose [3].

We and others have suggested that these results are mediated by the preferential and unregulated metabolism of fructose in the liver [4,5,6,7]. Hepatic fructose overload leads to upregulated DNL [3,8,9,10], reduced fat oxidation [8,9,11], and increased liver fat content [8,9,12], which are associated with increased synthesis and secretion of TG-rich VLDL_1_ (very low density lipoprotein) [13]. At high concentrations, VLDL_1_ becomes the favored substrate of cholesteryl ester transfer protein (CETP) [14] that catalyzes lipid transfer between lipoproteins. This leads to TG enrichment of LDL. TG-enriched LDL particles are the preferential substrate for the lipolytic action of hepatic lipase, which leads to smaller, denser particles [15]. However, whether sustained fructose consumption causes an increase in large TRL or TG enrichment of LDL particles has not been determined. Furthermore, apoCIII has been implicated as a major mediator of the metabolic processes that increase CVD risk [16,17] by causing reduced lipoprotein flux through clearance pathways and increased flux through the lipolysis pathways that lead to sdLDL [17]. In support of this, it was recently reported that the increase in LDL particle size caused by a weight loss intervention and the decrease in LDL particle size caused by a high-carbohydrate (32.5% of energy as complex, 32.5% as simple) dietary intervention, were both inversely correlated to the changes in apoCIII concentrations [18]. While it has been shown that consumption of both fructose [8,19] and fructose-containing sugar [20] leads to increased plasma apoCIII concentrations, it is not known if this effect is general for all carbohydrates or specific to fructose. Therefore, our objective was to determine the effects of sustained consumption of fructose-sweetened compared with glucose-sweetened beverages on fasting and postprandial circulating apoCIII and the TG-enrichment of large lipoproteins and LDL. We analyzed apoCIII concentrations and the TG and chol content of 20 lipoprotein fractions separated by size in fasting and postprandial plasma collected before and after intervention from subjects who consumed glucose- or fructose-sweetened beverages for 10 weeks [3].

## 2. Experimental Section

**Figure 1:**
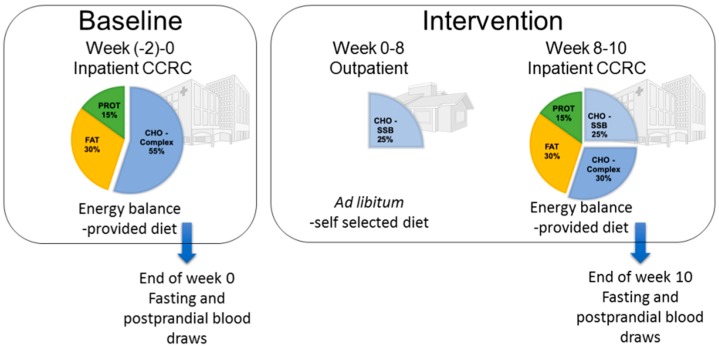
Study design and dietary protocol. CCRC: Clinical and Translational Science Center’s Clinical Research Center.

As previously reported [3], this was a matched, parallel-arm, dietary intervention study that consisted of three phases: (1) a two-week inpatient baseline period during which the subjects consumed an energy-balanced diet; (2) an eight-week outpatient intervention period during which the subjects consumed 25% of daily energy requirement as either glucose- (*n* = 15) or fructose-sweetened (*n* = 17) beverages, divided into three servings, along with their usual ad libitum diet; and (3) a two-week inpatient intervention period during which the subjects consumed 25% of their daily energy requirement as the assigned sugar-sweetened beverage along with an energy-balanced diet (Figure 1). Daily energy requirement was calculated by the Mifflin equation ([21]), with an adjustment of 1.3 for the days of the 24 h blood collections and an adjustment of 1.5 for the other days. Subjects resided in the University of California, Davis, (UCD), Clinical and Translational Science Center’s Clinical Research Center (CCRC) during the two-week baseline and two-week intervention inpatient periods of the study (Figure 1). Energy-balanced breakfast accounted for 25% of the subjects’ energy requirement, lunch for 35%, and dinner for 40%. The baseline diet consisted of 55% of energy as mainly complex carbohydrate, 30% fat, and 15% protein. Intervention meals mimicked the respective baseline meals in all but the carbohydrate composition, which consisted of 30% complex carbohydrate and 25% glucose- or fructose-sweetened beverages. During the eight-week outpatient intervention period, the subjects were instructed to drink three servings of the assigned beverages, one with each meal, and to refrain from drinking other sugar-containing beverages including fruit juices. We have previously reported that during the eight-week outpatient period, both groups gained comparable amounts of body weight (approximately 1.4 kg) [3].

Subjects: Participants were recruited through newspaper advertisements and underwent a telephone and an in-person interview with medical history, a complete blood count, and a serum biochemistry panel to assess eligibility. Inclusion criteria included age 40–72 years and BMI 25–35 kg/m^2^, with a self-report of stable body weight during the prior six months. Women were post-menopausal on the basis of a self-report of no menstruation for at least one year. Exclusion criteria included: evidence of diabetes, renal or hepatic disease, fasting serum TG concentrations >400 mg/dL, hypertension (>140/90 mg Hg), and surgery for weight loss. Also excluded were individuals who smoked, reported exercise of more than 3.5 h/week at a level more vigorous than walking, or having used thyroid, lipid-lowering, glucose-lowering, anti-hypertensive, anti-depressant, or weight loss medications. Diet-related exclusion criteria included habitual consumption of more than one sugar-sweetened beverage/day or more than two alcoholic beverages/day. All experimental procedures were in accordance with the Helsinki Declaration and approved by the UCD Institutional Review Board. All subjects provided informed written consent to participate in the study. Thirty-nine subjects enrolled in the study, and experimental groups were matched for gender, BMI, and fasting TG and insulin concentrations. Seven subjects (three in the glucose group, four in the fructose group) failed to complete the study due to inability/unwillingness to comply with the protocol or due to personal or work-related conflicts. The baseline anthropometric and metabolic parameters of the subjects were previously reported [3] and were equal between the experimental groups. The mean age, BMI, and baseline fasting plasma TG concentration of all subjects was 53.7 ± 1.4 years, 30.8 ± 1.0 kg/m^2^, and 145.2 ± 12.3 mg/dL, respectively.

After 10 days of energy-balanced feeding, 24 h serial blood collections were conducted during baseline (0 week) and the 10th week of intervention (10 week). Meals were served at 9:00 a.m., 1:00 p.m. and 6:00 p.m. The plasma from the three fasting samples (8:00 a.m., 8:30 a.m., 9:00 a.m.) was pooled, as was the plasma from three postprandial blood samples (10:00 p.m., 11:00 p.m., 11:30 p.m.). We chose 10:00–11:30 p.m. as the postprandial time-points because it was during this period that fructose had the most marked effects on TG concentrations compared with glucose during our previous study [22]. The 0-week and 10-week fasting and postprandial plasma samples from 31 of the 32 subjects (insufficient plasma obtained from one subject in the fructose group) were classified and quantified for chol and TG concentrations in 20 subfractions by high-performance liquid chromatography at Skylight Biotech (LipoSEARCH; Skylight Bio-tech Inc., Akita, Japan) to examine the lipoprotein profiles by subclass [23,24,25]. The subfractions were termed TRLp1-7, LDLp1-6, and HDLp1-7, respectively, and were classified by particle diameter (Table 1). The results pertaining to the HDLp1-7 subfractions are not reported in this paper. Apolipoprotein CIII (apoCIII) was measured in the same pooled samples used to determine fasting (8:00, 8:30, 9:00 a.m.) and postprandial (10:00, 11:00, 11:30 p.m.) lipoproteins. The concentrations were assessed with a Polychem Chemistry Analyzer (PolyMedCo Inc., Cortlandt Manor, NY, USA) with reagents from MedTest DX.

The effects of 2-, 8- and 10-week glucose and fructose consumption on the plasma concentrations of fasting and postprandial TG and apoB100, and fasting total, LDL, high-density lipoprotein (HDL), and sdLDL-chol were previously reported [3].

**Figure 2:**
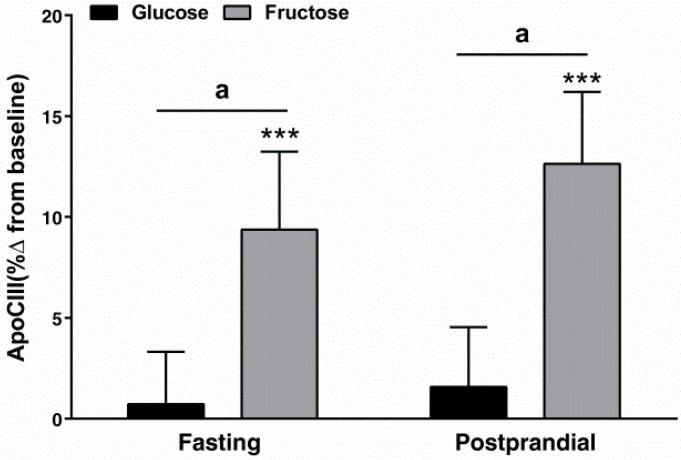
Percent (%) changes (10 weeks vs. 0 weeks) of apoCIII in the serum of subjects consuming glucose- (*n* = 15) or fructose-sweetened beverages (*n* = 16) for 10 weeks. ^a^ *p* < 0.05 effect of sugar, least squares (LS) means different from zero; *** *p* < 0.001, LS means different from zero—change from baseline. Data shown as mean ± SEM.

**Figure 3:**
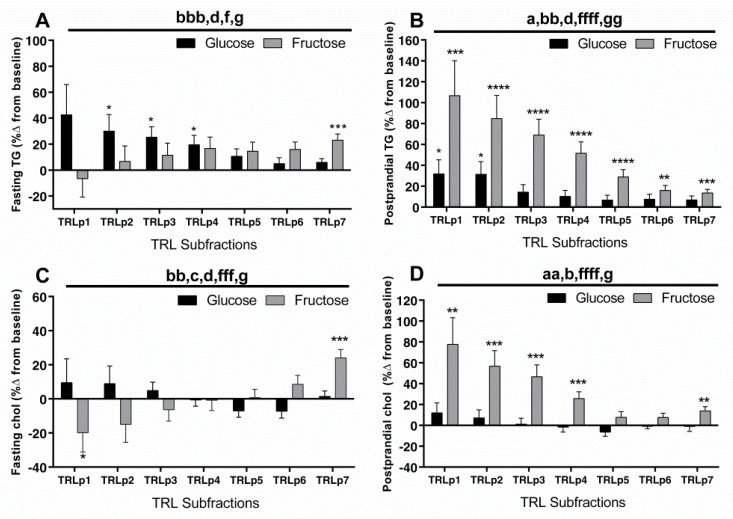
Percent (%) changes (10 weeks vs. 0 weeks) of fasting (**A**) and postprandial (**B**) TG and fasting (**C**) and postprandial (**D**) chol in TRL subfractions (chylomicrons (CM) and very low density lipoprotein (VLDL)) in subjects consuming glucose- (*n* = 15) or fructose-sweetened beverages (*n* = 16) for 10 weeks. ^a^ *p* < 0.05, ^aa^ *p* < 0.01, effect of sugar; ^b^ *p* < 0.05, ^bb^ *p* < 0.01, ^bbb^ *p* < 0.001, effect of particle size, ^c^ *p* < 0.05, effect of gender, ^d^ *p* < 0.05, effect of sugar x size; ^f^ *p* < 0.05, ^fff^ *p* < 0.00, ^ffff^ *p* < 0.0001 for fructose-induced lineal trend, ^g^ *p* < 0.05, ^gg^ *p* < 0.01 for glucose-induced lineal trend. * *p* < 0.05, ** *p* < 0.01, *** *p* < 0.001, **** *p* < 0.0001, LS means different from zero—within-group change from baseline. Data shown as mean ± SEM. Note the differences in scales.

**Figure 4:**
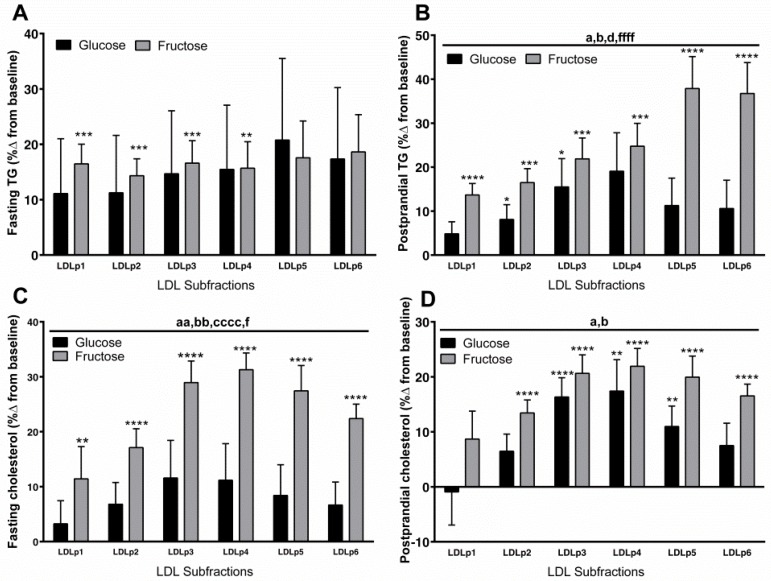
Percent (%) changes (10 weeks vs. 0 weeks) of fasting (**A**) and postprandial (**B**) TG and fasting (**C**) and postprandial (**D**) chol in LDL subfractions in subjects consuming glucose-sweetened beverages (*n* = 15) or fructose-sweetened beverages (*n* = 16) for 10 weeks. ^a^ *p* < 0.05, ^aa^ *p* < 0.01, effect of sugar; ^b^ *p* < 0.05, ^bb^ *p* < 0.01, effect of particle size; ^cccc^ *p* < 0.0001, effect of gender, ^d^ *p* < 0.05, effect of sugar × size, ^f^ *p* < 0.05; ^ffff^ *p* < 0.0001 for fructose-induced lineal trend, ^g^ *p* < 0.05 for glucose-induced lineal trend. * *p* < 0.05, ** *p* < 0.01, *** *p* < 0.001, **** *p* < 0.0001, LS means different from zero—within group change from baseline. Data shown as mean ± SEM. Note the differences in scales.

Statistical Analysis: Differences in the percent changes (delta Δ) in the TRL, LDL fractions (Table 2), and apoCIII (Figure 2) were analyzed with a generalized linear two-factor (sugar and gender) method. The percent changes of chol and TG in TRL (chylomicron, VLDL) and LDL subfractions at 10 weeks compared to baseline (Figure 3 and Figure 4) were analyzed by three-factor (sugar, subfraction size, gender), mixed procedures (PROC MIXED) repeated measures (subfraction size) ANOVA (SAS 9.4). Significant within-group changes from baseline for the individual subfractions were identified by least-squares means (LS means) of the percent changes significantly different from zero. Trend contrasts were used to identify linear relationships between particle size and glucose or fructose consumption. The symbols designating a significant effect of ANOVA factors are consistent for Figure 2, Figure 3 and Figure 4: a = sugar, b = particle size, c = gender, d = sugar × size, f = fructose-induced linear trend, g = glucose-induced linear trend. Pearson’s correlation coefficients were calculated for the changes of total postprandial TG, total and subfraction TRL TG, fasting and postprandial apoCIII, and total fasting LDL and LDLp3-6 chol (SAS 9.4). The data are presented as mean ± SEM.

## 3. Results

Figure 2 shows the percent changes of plasma apoCIII concentrations after glucose or fructose intervention. Fasting and postprandial apoCIII levels increased in subjects consuming fructose compared with subjects consuming glucose (*p* < 0.05 for both fasting and postprandial, effect of sugar).

The baseline fasting and postprandial contents of chol and TG were not significantly different between the groups in any of the TRL or LDL subfractions (Table 1). The baseline and intervention values and percent changes in the overall TRL and LDL fractions are shown in Table 2. The subjects consuming fructose had increased postprandial levels of chol and TG in both overall particle fractions. In addition, chol and TG were increased in fasting LDL fractions after fructose consumption. In the glucose group, fasting TRL TG and postprandial LDL TG and chol levels increased after the intervention compared to baseline. The fructose-induced increases were significantly higher than those induced by glucose for postprandial TG in TRL, postprandial chol in TRL and LDL, and fasting chol in LDL.

The percent changes of TG and chol (week 10 compared to baseline) in the TRL subfractions are shown in Figure 3. The two sugars induced opposite linear trends for the changes of fasting TG within the TRL subfractions (Figure 3A: *p* < 0.05, sugar × size; *p* < 0.05, both fructose and glucose linear trend). The subjects consuming glucose had increased TG content in the larger TRL particles (TRLp2–4), while those consuming fructose had increased TG content only in the smallest particles (TRLp7). The same opposing linear trends occurred for fasting chol in TRL (Figure 3C: *p* < 0.001, fructose linear trend; *p* < 0.05, glucose linear trend). In the postprandial state, the subjects consuming fructose had increased TG content in all TRL subfractions, with the highest changes in the largest TRL subfractions. This increase exhibited a highly significant linear trend (Figure 3B; *p* < 0.0001, fructose linear trend) in the opposite direction of the fasting trend. The effects of glucose consumption on postprandial TG content in the TRL subfractions were significantly lower, (*p* < 0.05, effect of sugar; *p* < 0.05 effect of sugar x size), with only TRLp1 and 2 showing a significant increase. The postprandial changes in TRL chol content (Figure 3D) paralleled the changes in TRL TG. The increases induced by fructose showed the same linear trend (*p* < 0.0001 for linear trend) and were higher than those induced by glucose (*p* < 0.01, effect of sugar).

The percent change at 10 weeks compared with baseline of fasting and postprandial chol and TG in the six LDL subfractions are shown in Figure 4. The fructose-induced increases of fasting TG content in LDL were comparable among the subfractions and were not significantly higher than those induced by glucose (Figure 4A). In contrast to the fasting state (Figure 4A), the fructose-induced increases in postprandial LDL TG were higher than those induced by glucose (*p* < 0.05, effect of sugar, effect of sugar x size) and displayed a highly significant linear trend with higher increases in the smaller particles (Figure 4B; *p* < 0.0001). Compared with glucose, fructose consumption significantly increased fasting chol content in the LDL subfractions, especially in the smaller subfractions (small dense (sd)LDL) (Figure 4C; *p* < 0.01, effect of sugar). The changes were higher in men than in women (Figure 4C; *p* < 0.0001, effect of gender). In the postprandial state, both sugars increased LDL TG and chol content, but the increases were higher and more significant in subjects consuming fructose than in subjects consuming glucose (Figure 4B-TG: *p* < 0.05, effect of sugar; *p* < 0.05, effect of sugar × size; Figure 4D-chol: *p* < 0.05, effect of sugar).

In order to compare the relationships of postprandial TG-rich particles and apoCIII to fasting sdLDL-chol, we performed regression analysis. Table 3 lists the regression coefficients and *p*-values for the relationships between the changes of fasting and postprandial apoCIII, total TG, total TLR TG, and TG in each TRL subfraction and the changes of total fasting LDL chol and fasting chol in the small LDL particles (LDLp3–6). In subjects consuming fructose, postprandial apoCIII correlated with total fasting LDL-chol (*p* < 0.03), while the individual TRL subfractions and total TG did not. There were significant associations (all *p* < 0.05) between the increase in fasting LDLp3–6 chol (sdLDL-chol) and the increase in postprandial apoCIII and postprandial TG in TRLp2 and TRLp3 in subjects consuming fructose, but not in subjects consuming glucose. In multivariate regression that included both postprandial apoCIII and TRLp2 or TRLp3, the significance of both were attenuated (apoCIII and TRLp2 *p* = 0.16 and *p* = 0.13; apoCIII and TRLp3 *p* = 0.20 and *p* = 0.20). There were significant positive correlations between postprandial apoCIII and total TRL TG (*p* < 0.0001) and between postprandial apoCIII and TRLp2 or TRLp3 TG (*p* < 0.05 for both).

## 4. Discussion

In the present study, we explored differences between circulating apoCIII and the TG and chol composition of lipoprotein fractions in subjects consuming glucose- or fructose-sweetened beverages for 10 weeks. The changes in apoCIII and in the patterns of TG and chol within the different lipoprotein fractions varied markedly between the two groups, despite their consuming standardized inpatient diets for 10 days prior to both baseline and 10-week intervention blood collections. The intervention diets differed solely in the composition of the beverages, specifically, in the type of added sugar, i.e., glucose or fructose.

ApoCIII is associated with increased CVD risk through various mechanisms, and several studies suggest it to be a robust and reliable predictor of CVD risk [26,27]. Our data show fasting and postprandial apoCIII levels increased after fructose consumption compared with the levels after glucose consumption. To the best of our knowledge, this is the first study to report increased responses of circulating apoCIII to fructose consumption compared with glucose consumption. The results suggest that the previously reported increases of plasma apoCIII concentrations in human subjects consuming fructose [8] or fructose-containing sugar [20] are specific to fructose rather than to carbohydrate in general. A possible explanation for these results may involve insulin, which is a negative transcriptional regulator of apoCIII expression [28]. We have previously reported that the two sugars had highly significant and opposite effects on circulating insulin, with glucose consumption increasing, and fructose consumption decreasing 24 h area under the curve (AUC) and post-meal insulin responses [29]. Cell culture experiments showed apoCIII expression is induced by glucose via hepatocyte nuclear factor 4 alpha (HNF-4α) and carbohydrate-responsive element-binding protein (ChREBP) [30] and is reduced by insulin via Forkhead Box O1 (FOXO1) [28]. This regulation takes place in the liver but not in the intestine, which are the two main sites of apoCIII expression [31]. Thus, it is possible that the failure of glucose consumption to increase circulating apoCIII is due to insulin’s negative feedback on hepatic apoCIII transcription. Fructose also activates ChREBP [32,33]. This activation, in the absence of negative feedback by insulin on apoCIII transcription [34], may explain the increased levels of circulating apoCIII after fructose consumption compared with glucose consumption.

It has been recently reported that apoCIII is the strongest predictor of hypertriglyceridemia in a large cohort of rhesus primates and that inhibition of apoCIII by RNA interference lowered fructose-induced hypertriglyceridemia [35]. ApoCIII may affect the lipid metabolism by promoting hepatic DNL and VLDL_1_ production [27,34,36,37,38,39,40] and by interfering with hepatic clearance of TRL through masking apoB/apoE receptors [16,17,41,42]. Both processes lead to increased and sustained TRL levels in the circulation, which are associated with CVD development and progression [43,44]. Therefore, to investigate the effects of fructose on the fasting and postprandial levels of TRL and other indicators of CVD risk, we measured TG and chol in lipoprotein particles separated by size. The subjects consuming glucose had increased fasting TG in large TRL particles, while those consuming fructose had increased fasting TG in small TRL particles. Thus, if our study only investigated the changes that occurred in the fasting state, these results could lead to the suggestion that consumption of glucose is associated with CVD risk to a larger extent than consumption of fructose. However, the postprandial changes induced by the two sugars in the TG content of the TRL subfractions differed dramatically from the changes in the fasting state with regard to the direction of the linear trend, the magnitude of the increases, and the differential effects of the beverages. Overall, they clearly demonstrate that, compared with glucose consumption, the consumption of fructose increased postprandial TG content in all TRL subfractions, with the increases being most marked in the largest particles. Given that people spend up to 18 h per day in a nonfasted state [45], these results from samples collected postprandially are likely to be more relevant to CVD risk than the fasting results. Furthermore, epidemiology studies provide evidence that non-fasting TG is a more reliable index of CVD risk than fasting TG [46,47]. Compared with glucose, fructose also increased postprandial chol content in the TRL subfractions, with the increases being most marked in the largest particles. This too may promote CVD risk. A prospective study on 90,000 individuals showed a dose-dependent effect of non-fasted remnant cholesterol on later ischemic heart disease and myocardial infarction [48].

The prominently increased postprandial TRL TG and chol in the subjects consuming fructose may result from impaired TRL clearance or increased TRL synthesis—or a combination of both. As stated above, apoCIII could be involved in TRL clearance and/or increased TRL synthesis. Other possible mechanisms not involving apoCIII include a direct effect of fructose overload on the upregulation of hepatic DNL. We have previously reported that postprandial hepatic DNL was significantly increased in the subjects consuming fructose compared to those consuming glucose [3]. Also, it has been suggested, although the available evidence to date is limited, that disruption of enterocyte lipid metabolism may make a meaningful contribution to the hypertriglyceridemia often associated with fructose consumption [49,50]. Fructose feeding has been shown to increase chylomicron synthesis in enterocytes via upregulated DNL and reduced apoB48 degradation in a hamster model of insulin resistance [49]. Impaired TRL clearance could be mediated by decreased lipoprotein lipase (LPL) activity [3], which catalyzes the lipolysis of TG from TRL in the circulation. ApoCII is an important cofactor for LPL activation [51,52] and could be involved in the in differential effects of the two beverages on TRL clearance; however, the effects of fructose compared with those of glucose on apoCII have yet to be investigated.

We previously reported that sdLDL cholesterol was increased in the subjects consuming fructose compared with those consuming glucose [3], and the current data confirm this. The high levels of apoCIII may be involved in generating sdLDL by inhibiting lipoprotein clearance pathways and promoting the lipolytic conversion of TRL, IDL, and LDL to smaller, denser LDL particles [17]. However, the traditional view on the generation of sdLDL involves cholesteryl ester transfer protein (CETP) -mediated TG transfer from TRL to LDL [15,53,54]. Supportive of this, our results showed an increase in the TG content of all LDL subfractions during the postprandial period after fructose consumption compared to glucose consumption. TG-enriched LDL has reduced affinity for the LDL receptor and a longer residence time in the circulation compared to LDL with normal TG content [55]. It is therefore exposed to hepatic lipase, which lyses TG. Accordingly, the LDL particles from subjects consuming fructose were less enriched with TG in the fasting state than in the postprandial state (*p* < 0.001 for all individual LDL subfractions, paired t tests). At the same time, these fasting particles had increased cholesterol content compared to the LDL particles from subjects consuming glucose, especially in the smaller particles (LDLp3-6).

Regression analyses showed associations between the changes in fasting sdLDL-chol and postprandial apoCIII and large particle TRLs (TRLp2 and TRLp3) only in subjects consuming fructose. The results are in agreement with the hypothesis that increased and sustained concentrations of large TRL particles lead to lipoprotein changes that result in the formation of sdLDL-chol [15,56,57] and the possibility that apoCIII has a major role in mediating these metabolic processes [17]. A recent intervention trial showed apoCIII was positively associated with sdLDL formation after a high-carbohydrate diet that contained equal amounts of complex and simple carbohydrate [18]. Here, we expand on these results showing that the elevation of apoCIII and its association with sdLDL formation occurred after fructose, but not glucose, consumption. The results showing that apoCIII also correlated with the changes in total LDL-chol may suggest that apoCIII impairs clearance of all LDL particles. In contrast, neither TRLp2 nor TRLp3 correlated with total LDL-chol. Possibly, the effects of TRLp2 and TRLp3 were more specific to LDLp3–6 because they mediated higher TG-enrichment in LDLp3-6 than in LDLp1 (*p* = 0.003–0.04) or p2 (*p* = 0.003–0.06, all comparisons, paired *t* tests)). However, the attenuated effects of both apoCIII and TRLp2 or TRLp3, when included in the same multi-regression analysis, suggests that their effects on the increase in fasting chol in LDLp3–6 are mediated by dependent pathways.

Elevated levels of sdLDL have been described as independent predictors of cardiovascular events in patients with non-coronary atherosclerosis [58,59,60] and also of cardio- and cerebro-vascular events in patients with metabolic syndrome [59]. Increased sdLDL, along with elevated levels of TRL, LDL cholesterol, oxidized LDL, and apoB and low levels of HDL-chol constitute the ‘atherogenic dyslipidemia complex’, a feature of type 2 diabetes and the metabolic syndrome [61]. The subjects consuming fructose-sweetened beverages for 10 weeks exhibited adverse changes in all components of the ‘atherogenic dyslipidemia complex’, excepting lowered HDL-chol concentrations. As previously reported, plasma HDL concentrations were unchanged at 10 weeks in the subjects consuming fructose- or glucose-sweetened beverages [3].

A limitation to our study is the selective inclusion of older and overweight subjects, which may limit our findings to this group. However, as this demographic is increasing and already at a high risk for CVD, our reported findings are valuable even if younger and healthier subjects react differently to sugar consumption. The modest sample size limited the exploration of gender effects, which should be studied further with increased subject numbers. Finally, this study does not investigate the effects of sugar-sweetened beverage consumption as they are commonly consumed in this country, with regard to both the amount of sugar consumed and the types of sugars consumed. Self-reported intake data suggest that only 13% of the US population consumes >25% of energy from added sugars (41), and the majority of the added sugar is not pure fructose or glucose, but rather high fructose corn syrup (HFCS) (55% fructose, 45% glucose) and sucrose (50% fructose, 50% glucose). However, the study of fructose and glucose separately allowed us to demonstrate that fructose increases circulating apoCIII compared to glucose, thus, it is the likely mediator of the increases in apoCIII induced by a high-carbohydrate diet [18] or HFCS-sweetened beverages [20].

Furthermore, mechanistic insights gleaned from investigations of fructose compared to glucose are relevant to explaining the observed increases in postprandial TG, fasting and/or postprandial apoCIII, LDL-chol, sdLDL, and apoB observed in subjects consuming HFCS or sucrose-sweetened beverages [12,20,62,63].

## 5. Conclusions

The results from this study demonstrate that consumption of fructose increases fasting and postprandial plasma concentrations of apoCIII compared with the consumption of glucose and support the involvement of apoCIII in the development of sdLDL and CVD risk [17]. The results also show that fructose markedly increases large TRL particles and the TG-enrichment of LDL in the late postprandial period, which may also affect the development of sdLDL and CVD risk [3,15,56,57]. As the adverse effects of fructose compared with glucose occurred after 10 days of controlled dietary conditions, the results do not support the often-repeated belief that “a calorie is a calorie” independent of its source. While more research is required to determine the levels of fructose-containing sugar that can be consumed without increased risk, it is prudent to advise patients at risk for CVD to refrain from drinking beverages sweetened with fructose-containing sugars.

## Figures and Tables

**Table 1:** Baseline values of fasting and postprandial cholesterol (chol) and triglycerides (TG) in TG-rich lipoproteins (TRL) and low-density lipoprotein (LDL) subfractions.

	Diameter	Cholesterol (mg/dL)				TG (mg/dL)			
		Fasting		Postprandial		Fasting		Postprandial	
	(nm)	Glucose	Fructose	Glucose	Fructose	Glucose	Fructose	Glucose	Fructose
TRLp1	>90	2.7 ± 0.6	3.2 ± 0.8	3.4 ± 0.7	3.6 ± 0.9	10.0 ± 2.6	14.1 ± 3.4	19.0 ± 4.1	22.1 ± 5.0
TRLp2	75	1.4 ± 0.2	1.5 ± 0.3	1.6 ± 0.2	1.6 ± 0.3	6.6 ± 1.3	6.9 ± 1.3	10.1 ± 1.7	10.3 ± 2.1
TRLp3	64	3.8 ± 0.5	3.7 ± 0.5	4.1 ± 0.5	3.7 ± 0.6	14.9 ± 2.6	14.1 ± 2.3	18.4 ± 2.7	17.9 ± 2.9
TRLp4	53.6	7.4 ± 0.7	6.8 ± 0.7	7.1 ± 0.7	6.4 ± 0.8	27.1 ± 4.0	23.9 ± 3.4	29.7 ± 4.0	28.0 ± 4.0
TRLp5	44.5	16.5 ± 1.0	14.6 ± 1.0	14.4 ± 1.2	12.6 ± 1.1	33.9 ± 4.4	28.9 ± 3.5	35.0 ± 4.3	32.7 ± 3.9
TRLp6	36.8	12.9 ± 1.1	11.5 ± 1.2	10.6 ± 1.4	9.5 ± 1.3	17.7 ± 2.0	15.1 ± 1.7	18.5 ± 2.0	17.6 ± 1.9
TRLp7	31.3	6.0 ± 0.4	5.5 ± 0.6	6.4 ± 0.5	6.0 ± 0.5	5.0 ± 0.5	4.3 ± 0.5	5.6 ± 0.5	5.5 ± 0.5
LDLp1	28.6	19.4 ± 1.1	18.2 ± 1.2	20.7 ± 1.6	19.1 ± 1.2	7.6 ± 0.6	6.9 ± 0.7	8.7 ± 0.7	8.5 ± 0.7
LDLp2	25.5	39.0 ± 1.3	36.3 ± 2.1	36.6 ± 1.6	35.7 ± 2.1	8.4 ± 0.6	8.0 ± 0.8	9.4 ± 0.7	9.6 ± 1.0
LDLp3	23.0	21.7 ± 1.3	20.2 ± 1.9	19.0 ± 1.6	18.6 ± 1.7	5.5 ± 0.5	5.1 ± 0.6	5.9 ± 0.6	6.0 ± 0.8
LDLp4	20.7	6.3 ± 0.5	5.8 ± 0.6	5.6 ± 0.7	5.2 ± 0.5	2.1 ± 0.2	1.9 ± 0.2	2.2 ± 0.3	2.2 ± 0.3
LDLp5	18.6	2.5 ± 0.2	2.4 ± 0.2	2.3 ± 0.2	2.2 ± 0.2	1.1 ± 0.1	1.1 ± 0.1	1.4 ± 0.2	1.3 ± 0.2
LDLp6	16.7	1.4 ± 0.1	1.3 ± 0.1	1.3 ± 0.1	1.2 ± 0.1	0.7 ± 0.1	0.7 ± 0.1	1.0 ± 0.1	0.9 ± 0.1

Mean ± SEM.

**Table 2:** Total fasting (FST) and postprandial (PP) TG and cholesterol concentrations in TRL and LDL fractions before and after consumption of glucose- and fructose-sweetened beverages for 10 weeks.

	Glucose			Fructose		
	0 weeks	10 weeks	% change	0 weeks	10 weeks	% change
**Lipoprotein TG (mg/dL)**						
TRL TG–FST	116.4 ± 17.3	122.8 ± 16.6	14.3 ± 6.0 *	107.3 ± 14.5	111.9 ± 15.4	7.2 ± 6.8
TRL TG–PP	138.3 ± 19.4	149.8 ± 18.9	11.4 ± 6.1	134.2 ± 18.8	180.9 ± 22.4	42.9 ± 8.3 ^aa,^****
LDL TG–FST	24.7 ± 2.3	26.1 ± 2.2	6.3 ± 6.3	23.7 ± 2.5	26.8 ± 2.9	13.9 ± 5.3 ****
LDL TG–PP	27.3 ± 2.7	30.6 ± 2.2	8.8 ± 4.4 *	28.5 ± 2.9	33.2 ± 3.1	18.6 ± 3.1 ****
**Lipoprotein Chol (mg/dL)**						
TRL Chol–FST	51.3 ± 43.7	46.3 ± 5.3	−4.1 ± 3.0	46.7 ± 4.0	47.9 ± 4.7	2.6 ± 3.8
TRL Chol–PP	48.1 ± 4.3	46.4 ± 6.1	−2.7 ± 3.6	43.4 ± 4.5	49.8 ± 4.7	16.3 ± 5.1 ^aa,^***
LDL Chol–FST	90.4 ± 3.3	96.1 ± 3.9	7.0 ± 3.7	84.3.0 ± 5.3	101 ± 7	19.3 ± 2.9 ^a,^****
LDL Chol–PP	86.0 ± 3.9	90.3 ± 3.4	7.1 ± 2.6 **	82.1 ± 5.0	94.3 ± 6.1	14.7 ± 1.9 ^a,^****

^a^*p* < 0.05, ^aa^ *p* < 0.01, effect of sugar. * *p* < 0.05, ** *p* < 0.01, *** *p* < 0.001, **** *p* < 0.0001, LS mean of % change different than zero. Mean ± SEM.

**Table 3:** The relationship of percent change of postprandial total, TRL subfraction TG, and fasting and postprandial apoCIII to the absolute increase of total fasting LDL and LDLp3–6 cholesterol.

	Total FST LDL Cholesterol				FST LDLp3-6 Cholesterol			
	Glucose	*P* Value	Fructose	*P* Value	Glucose	*P* Value	Fructose	*P* Value
	*r*		*r*		*r*		*r*	
Total TG–PP	0.03	0.91	0.21	0.44	−0.13	0.65	0.21	0.43
Total TRL TG–PP	0.04	0.88	0.01	0.97	−0.14	0.64	0.28	0.29
TRLp1 TG–PP	−0.07	0.82	−0.08	0.77	−0.21	0.47	0.40	0.12
TRLp2 TG–PP	0.02	0.94	−0.04	0.88	−0.23	0.42	0.53	**0.04**
TRLp3 TG–PP	0.10	0.72	0.01	0.96	0.03	0.91	0.51	**0.05**
TRLp4 TG–PP	0.06	0.83	0.07	0.78	−0.04	0.88	0.39	0.14
TRLp5 TG–PP	0.08	0.78	0.06	0.84	−0.09	0.75	0.33	0.21
TRLp6TG–PP	0.04	0.88	−0.17	0.53	−0.14	0.63	0.16	0.54
TRLp7 TG–PP	−0.07	0.82	−0.27	0.30	−0.22	0.44	−0.06	0.82
ApoCIII FST	−0.27	0.36	0.44	0.09	−0.37	0.20	0.44	0.09
ApoCIII PP	−0.32	0.26	0.53	**0.03**	−0.48	0.08	0.51	**0.04**

*r*: Pearson’s correlation coefficient; FST: fasting state; PP: postprandial state. Bold: indicates significance (*p* < 0.05).
